# Algorithmic Content Recommendations on a Video-Sharing Platform Used by Children

**DOI:** 10.1001/jamanetworkopen.2024.13855

**Published:** 2024-05-29

**Authors:** Jenny Radesky, Enrica Bridgewater, Shira Black, August O’Neil, Yilin Sun, Alexandria Schaller, Heidi M. Weeks, Scott W. Campbell

**Affiliations:** 1 Department of Pediatrics, University of Michigan Medical School, Ann Arbor; 2 Department of Communication & Media, University of Michigan, Ann Arbor; 3 Department of Nutritional Sciences, University of Michigan School of Public Health, Ann Arbor; 4 Department of Psychology, University of Michigan, Ann Arbor

## Abstract

**Importance:**

Free video-sharing platforms (VSPs) make up a high proportion of children’s daily screen use. Many VSPs make algorithmic recommendations, appearing as thumbnail images from the video, which content creators use to advertise their video content.

**Objective:**

To explore how VSP thumbnails use attention-capture designs to encourage engagement with content and to test whether VSP algorithmic recommendations offer more problematic thumbnail features over time.

**Design, Setting, and Participants:**

In this cross-sectional study conducted in January 2022, researchers mimicked the search behavior of children on a popular VSP by randomly clicking on recommended videos in order to test whether thumbnail designs changed over 20 sequential video engagements. A digital, footprint-free data collection setting was created by using a new computer and wireless internet router. Data were collected from YouTube via an internet browser not logged into a user account. Data analysis occurred from April to December 2022.

**Exposures:**

Manual searches using 12 top-searched terms popular with school-aged children were conducted. Researchers captured the video thumbnails recommended at the end of each video and randomly clicked subsequent videos for 20 sequential engagements.

**Main Outcomes and Measures:**

Thumbnail content codes were developed through iterative review of screenshots by a multidisciplinary research team and applied by trained coders (reliability, κ >.70). The prevalence of problematic thumbnail content and change in prevalence over 20 engagements was calculated using the Cochran-Armitage trend test.

**Results:**

A total of 2880 video thumbnails were analyzed and 6 features were coded, including visual loudness; drama and intrigue; lavish excess and wish fulfillment; creepy, bizarre, and disturbing; violence, peril, and pranks; and gender stereotypes. A high proportion contained problematic features including the creepy, bizarre, and disturbing feature (1283 thumbnails [44.6%]), violence, peril, and pranks feature (1170 thumbnails [40.6%]), and gender stereotypes feature (525 thumbnails [18.2%]). Other features included attention-capture designs such as the visual loudness feature (2278 thumbnails [79.1%]), drama and intrigue feature (2636 thumbnails [91.5%]) and lavish excess and wish fulfillment feature (1286 thumbnails [44.7%]). Contrary to the hypotheses, problematic feature prevalence did not increase over time, but the gender stereotypes feature increased with more engagement in the recommendations feed (*P *for trend < .001).

**Conclusions and Relevance:**

In this study of video recommendations for search terms popular with children, thumbnails contained problematic and attention-capturing designs including violent, stereotyped, and frightening themes. Research is needed to understand how children respond to thumbnail designs and whether such designs influence the quality of content children consume.

## Key Points

**Question:**

What content is likely to be recommended to children on popular video-sharing platforms?

**Findings:**

In this cross-sectional study of 2880 video thumbnails from a popular video-sharing platform, searching with 12 search terms popular with children yielded recommended video thumbnails that contained a high prevalence of attention-engaging and problematic content such as violence or frightening images.

**Meaning:**

These findings suggest that video-sharing platforms may recommend problematic videos to children when they search for popular content.

## Teaser

This cross-sectional study examines how video-sharing platforms use attention-grabbing designs in video thumbnails to encourage engagement among children and whether recommendations offer more problematic content over time.

## Introduction

Video-sharing platforms (VSPs) are very popular among children. Recent estimates suggest that children 8 years and younger spend about 65% of their online time on video-sharing sites,^1^ many averaging over 1 hour per day.^2^ Hundreds of hours of videos are uploaded to VSPs every minute,^3^ so most content moderation relies on automated systems that classify whether a video violates that platform’s policies, for example, by depicting violent or dangerous content. In other words, humans do not review most videos posted on VSPs before they are viewed by children. As a result, many parents report that age-inappropriate videos have been recommended to their children^4^ (eg, disturbing videos featuring characters popular among children^5^).

In response to this problem, some platforms have created made-for-kids labels, indicating that the videos are intended for child audiences. While such labels are easily applied to early childhood content (eg, nursery rhyme videos for toddlers), recent research^6,7^ shows that many children 8 years and younger seek out influencer, video game, or humorous videos that lack a made-for-kids designation. This opens child viewers to recommendations that may not be age-appropriate due to violent, sexualized, or stereotyped content.^8^ When children consume age-inappropriate or violent media, they have a higher rate of disrupted sleep,^9^ externalizing behavior problems,^10^ and problematic media use.^11^

Automated recommender systems shape what content children see on VSPs but have received very limited study. These systems operate by incorporating information about what videos are trending (ie, have high engagement in the form of comments or likes) and what videos cluster in the same user patterns (ie, viewers who like cat videos) to generate a list of recommended videos to watch next. Because video creators earn more advertising revenue when they get more views,^12^ some creators use a design approach termed *clickbait* to capture viewers’ attention via video thumbnails^13^ (ie, small still images that advertise what will appear in videos). Clickbait tactics described in the literature include abnormally appearing objects, emotional drama, or surprising and/or misleading information.^14^ Despite children’s regular interaction with clickbait and thumbnails while navigating VSPs, no studies have examined this phenomenon. This is an important area of study because the design of both thumbnails and recommender systems are likely to have a strong role in shaping the content quality of children’s video viewing. Moreover, automated systems powered by artificial intelligence have been implicated in elevating problematic content^15^ and perpetuating human biases.^16,17^ Therefore, more needs to be known about their potential impact on children’s problematic media experiences.

The purpose of our study was to identify engagement tactics that children may encounter in VSP thumbnails and recommendation feeds. We focused on search terms popular in middle-childhood (ie, ages 6 to 11 years) because in this developmental window, children are more independent in their media use and often use VSPs, but rarely watch made-for-kids content.^6,8^ We hypothesized that the prevalence of problematic thumbnail features would increase over time with sequential engagement with recommended videos (ie, approximating going down the rabbit hole).

## Methods

This cross-sectional study was deemed exempt from review by the University of Michigan institutional review board because the study did not include human participants or require informed consent. The study followed the Strengthening the Reporting of Observational Studies in Epidemiology (STROBE) reporting guideline. We conducted an analysis of recommended video content by coding the appearance of video thumbnails using a novel coding scheme. We collected data on 12 search terms likely to be of interest to school-aged children but unlikely to be designated as made-for-kids and collected sequential screenshots of recommended content grids to examine whether the prevalence of different thumbnail features changed over the course of engaging with recommended videos. We tested an evidence-informed hypothesis, reducing potential sources of bias in data collection, and clearly defining our variables and analytic approach.

### Study Design and Data Collection

We collected data from YouTube, the most-used VSP among children.^4^ To reflect the most common viewing practices among school-aged children, we used a combination of the top searches of 2020^24^ and recent research analyzing VSP viewing histories of children.^6,8^ We removed search terms we determined to be less relevant to school-aged children (eg, *baby shark* and *Joe Rogan*) and musical artists who were not VSP content creators (eg, BTS or Billie Eilish). Search terms included *PewDiePie, Fortnite, DanTDM, Minecraft, MrBeast, FGTeeV, Flamingo, memes, unspeakable, try not to laugh, Roblox, *and *SML *(ie, Super Mario Logan).

To eliminate prior digital behavior traces that could influence recommendation feeds, we conducted data collection on a new, previously unused laptop computer connected to a previously unused Wi-Fi router. Searches were conducted in January 2022 on a VSP with autoplay disabled, logged out on an internet browser. The researcher manually entered each term into the search box and clicked on the first unsponsored video to appear on the results page. At the end of each video, the researcher saved a screenshot of the video recommendations grid in full screen.

Then, using a random number generator, the researcher generated a number from 1 to 12 that indicated which recommended video thumbnail to click on next (we designated thumbnail numbers starting at 1 in the upper left corner, counting left to right, with the lower right thumbnail being number 12). If the random number generator returned a number that corresponded to a video with a Mix+ designation, meaning it was a playlist that autoplays content, a new number was generated. The researcher continued to take screenshots of recommendation grids for a total of 20 screenshots per search term. This yielded a total of 240 screenshots, each containing 12 thumbnails. Of note, when designing the data collection protocol, we considered using an approach in which the researcher clicked upon the most visually salient thumbnail in the recommendations grid, but there were so many highly salient thumbnails that research team was unable to create reliable criteria for sampling by this method.

### Coding Scheme Development and Implementation

The thumbnail content coding scheme was developed based on iterative discussions as part of an undergraduate seminar that met weekly in the fall of 2021. Students were assigned videos and thumbnails to review and were asked to describe their impressions of salient visual features and attention-capture designs. Based on seminar discussions, prior research on attention-capture designs,^18^ and research describing heuristics in VSP content,^8^ a preliminary coding scheme comprising 8 features was developed. Students were then assigned thumbnails to code, and the scheme was iteratively reduced into 6 distinct feature codes around which consensus emerged. Each code was grouped into a coding level (0 = absent, 1 = mild or latent, and 2 = clear or dominant). Students were trained in biweekly meetings; we calculated their coding reliability by comparing it with a benchmark for 6 different screenshots (72 thumbnails). Three students with acceptable reliability (weighted κ >.70) coded the remainder of the thumbnails between January and September 2022. Coders met with the lead author (J.R.) weekly to review coding progress and uncertainties, which were resolved by consensus. To reduce coder bias, screenshot files were randomly named so that coders could not identify which search term or sequential screenshot they were coding. When thumbnails were identified as duplicates (ie, already coded on prior screenshot, [306 screenshots]), a consistent code was applied for all instances of that thumbnail.

### Statistical Analysis

First, we calculated the frequency and proportion of codes for each thumbnail content feature (ie, occurrence of codes of levels 0, 1, or 2) across all search terms, and then separately for each search term. We also examined Spearman correlations between content features within the same thumbnail to determine which features tended to cooccur. To examine whether the frequency of content features changed over the course of 20 sequential screenshots, we conducted Spearman correlations between screenshot number (1 through 20) and each feature. We also dichotomized each feature as present (codes of levels 1 or 2) vs not present and tested significance of trend over 20 screenshots with the Cochran-Armitage trend test. The threshold for statistical significance was a 2-sided *P* < .05. Data analysis was conducted from April to December 2022 using SAS software version 9.4 (SAS Institute).

## Results

Of the 2880 total thumbnails initially collected, 2574 unique video thumbnails were coded for attention-capture and problematic content features as described in Table 1. These design approaches leveraged color, text, object placement, faces with exaggerated emotional expressions, bodies, and objects that were often luxurious, satisfying, or scary. Presence of thumbnail content codes varied by search term as shown in the eTable 1 in Supplement 1. Examples of content are available in eTable 2 in Supplement 1.

**Table 1.** Description and Prevalence of Thumbnail Content in Recommended Videos^a^

Feature name	Prevalence, No. (%) (N = 2880)^b^		Description
	Level 1	Level 2	
Visual loudness	1352 (46.9)	926 (32.2)	Attention-grabbing visual design that used perceptually salient features such as bright or highly saturated colors, high density of characters, dark colors or high contrast, text in large letters with lots of exclamation points, or large faces with extreme facial expressions
Drama and intrigue	1606 (55.8)	1030 (35.8)	Images that conveyed a shocking, dramatic, or outrageous message that invites click-through to find out what is going on; this includes manufactured conflict (eg, versus thumbnail); facial expressions that are extreme, shocked, or disgusted; dramatic situations; and images that are intentionally vague, confusing, or altered or strange in a way that aims to arouse curiosity
Lavish excess and wish fulfillment	860 (29.9)	426 (14.8)	Depiction of luxury items such as cars, jewelry, houses, large amounts of food, or lots of money (any depiction of a large amount of currency, even if a negative value); this includes objects and experiences that viewers might not be able to experience in real life, such as watching an extensive videogame world being built, lots of toys or objects on display, unboxings, or expensive technology or items
Creepy, bizarre, and disturbing	842 (29.2)	441 (15.3)	Thumbnails include elements such as odd, distorted images (eg, cartoon faces, odd juxtapositions like faces on suitcases); depictions of frightening characters or objects; and skeletons, monsters, or coffins. Images also seemed to elicit disgust or leverage attraction to horror content
Violence, peril, and pranks	891 (30.9)	279 (9.7)	Thumbnails depict gore, injury, or violence; people appearing to prank one another or do dangerous activities; appearance of weapons or dead bodies; dangerous substances like lava; challenges that are perilous; and references to death or a threat of injury (eg, motor vehicle crashes)
Gender stereotypes	387 (13.4)	138 (4.8)	Sexual objectification to attract click-through including depictions of exaggerated male or female bodies, sexual content or innuendos, and idealized depictions of bodies in video games

^a^ Search terms included: *PewDiePie, Fortnite, DanTDM, Minecraft, MrBeast, FGteeV, Flamingo, memes, unspeakable, try not to laugh, Roblox, *and* SML*.

^b^ Level 1 indicates present to a lesser degree or latent and implied and level 2 indicates clearly present.

### Attention-Capture Designs

The visual loudness feature was characterized by perceptually salient characteristics such as bright and highly saturated colors (often red and green), which seemed to be brightened through photo editing filters. Visual loudness included thumbnails with high contrast between light and dark colors and/or text written in capital letters with exclamation points. Thumbnails could also be visually cluttered with bright objects or characters. This feature occurred as a level 1 or 2 in 2278 thumbnails (79.1%), with the highest proportion in the search terms *SML* (230 of 240 thumbnails [95.8%]) and *FGTeeV* (214 of 240 thumbnails [89.2%]).

The drama and intrigue feature was characterized by images that conveyed a shocking, dramatic, or outrageous message that invited click-through to find out what might happen in the video. This included expression of a conflict (eg, thumbnails that posted 1 image vs another); facial expressions that are amazed, shocked, or disgusted; or images that are intentionally vague, confusing, strange, or altered in a way that aims to arouse curiosity. Trends (eg, challenges and fads) were often used to drive this drama and curiosity, such as *Squid Game*. This feature occurred as a level 1 or 2 in 2636 thumbnails (91.5%), with the highest proportion in the search terms *Roblox* (218 of 240 thumbnails [96.7%]), *Minecraft* (228 of 240 thumbnails [95.0%]), and *FGTeeV* (228 of 240 thumbnails [95.0%]).

The lavish excess and wish fulfillment feature occurred when there was a depiction of luxury items (eg, cars, jewelry, houses, or junk food) or lots of money (any depiction of a large amount of money or bitcoin and currency, even a negative value), often combined with a challenge. This included objects and experiences that viewers might not be able to experience in real life, such as watching an extensive videogame world being built, lots of toys, satisfying items like slime or candy, or expensive technology or items (eg, rare virtual objects from video games). Thumbnails often included an excessive number of objects (eg, a ball pit filling up a school bus). This feature occurred as a level 1 or 2 in 1286 thumbnails (44.7%), with the highest proportion in the search terms *MrBeast* (146 of 240 thumbnails [60.8%]), *SML* (139 of 240 thumbnails [57.9%]), and *Roblox *(138 of 240 thumbnails [57.5%]).

### Problematic Content Features

The creepy, disturbing, and bizarre feature was characterized by odd, distorted images (eg, cartoon character faces and odd juxtapositions such as children’s faces on suitcases) or depictions of frightening characters or objects (eg, skeletons, monsters, and coffins). Images also seemed to elicit disgust or leverage attraction to horror content (eg, a stuffed animal hanging from a noose). This feature occurred as a level 1 or 2 in 1283 thumbnails (44.6%), with the highest proportion in the search terms *Flamingo* (190 of 240 thumbnails [79.2%]) and *Memes* (155 of 240 thumbnails [64.6%]).

The violence, peril, and pranks feature was characterized by depictions of blood, gore, injury, violence, or people appearing to prank one another or do dangerous activities. This included any appearance of weapons or dead bodies, dangerous substances like lava, guns, challenges that are perilous, and references to death or a threat of injury (eg, motor vehicle crashes). This feature occurred as a level 1 or 2 in 1170 thumbnails (40.6%), with the highest proportion in the search terms *Flamingo* (145 of 240 thumbnails [60.4%]) and *DanTDM* (115 of 240 thumbnails [47.9%]).

The gender stereotypes feature included sexual objectification to attract click-through. This included depictions of exaggerated male or female bodies, sexual innuendos, or idealized depictions of bodies in video games. This feature occurred as a level 1 or 2 in 525 thumbnails (18.2%), with the highest proportion in the search terms *Roblox* (101 of 240 thumbnails [42.1%]) and *Flamingo* (64 of 240 thumbnails [26.7%]).

Several thumbnail features had small to moderate positive correlations with one another, the largest being between the violence and pranks feature and drama and intrigue (ρ = 0.29), the violence, peril, and pranks feature and creepy, bizarre, and disturbing feature (ρ = 0.28), and the visual loudness feature and lavish excess and wish fulfillment feature (ρ = 0.21) (Table 2). There were also a few small to moderate negative correlations, the largest being between the creepy and bizarre feature and lavish excess and wish fulfillment feature (ρ = −0.25).

**Table 2.** Spearman Correlations Between Content Codes Among 2880 Thumbnails

Content code	Spearman correlation coefficient (*P* value)						
	Screenshot No. (1-20)	Visual loudness	Drama and intrigue	Creepy, bizarre, and disturbing	Violence, peril, and pranks	Gender stereotypes	Lavish excess and wish fulfillment
Screenshot No. (1-20)	1.0	NA	NA	NA	NA	NA	NA
Visual loudness	.09 (<.001)	1.0	NA	NA	NA	NA	NA
Drama and intrigue	−0.01 (.54)	0.18 (<.001)	1.0	NA	NA	NA	NA
Creepy, bizarre, and disturbing	0.01 (.73)	0.16 (<.001)	0.17 (<.001)	1.0	NA	NA	NA
Violence, peril, and pranks	0.02 (.38)	0.078 (<.001)	0.29 (<.001)	0.28 (<.0001)	1.0	NA	NA
Gender stereotypes	−0.08 (<.001)	0.08 (<.001)	0.12 (<.001)	0.02 (.35)	0.03 (.13)	1.0	NA
Lavish excess and wish fulfillment	0.02 (.29)	0.21 (<.001)	0.01 (.75)	−0.25 (<.001)	−0.17 (<.001)	0.05 (.01)	1.0

Abbreviation: NA, not applicable.

### Trends Over 20 Screenshots

Both Spearman correlations and tests of trend demonstrated that visual loudness increased over the 20 screenshots (P for trend < .001), while gender stereotypes decreased (*P *for trend < .0001) (Figure). Other features showed no significant trend (drama and intrigue, *P* for trend = .12; lavish excess and wish fulfillment, *P *for trend = .22; creepy, bizarre, and disturbing, *P *for trend = .63; violence and pranks, *P *for trend = .13).

**Figure.**
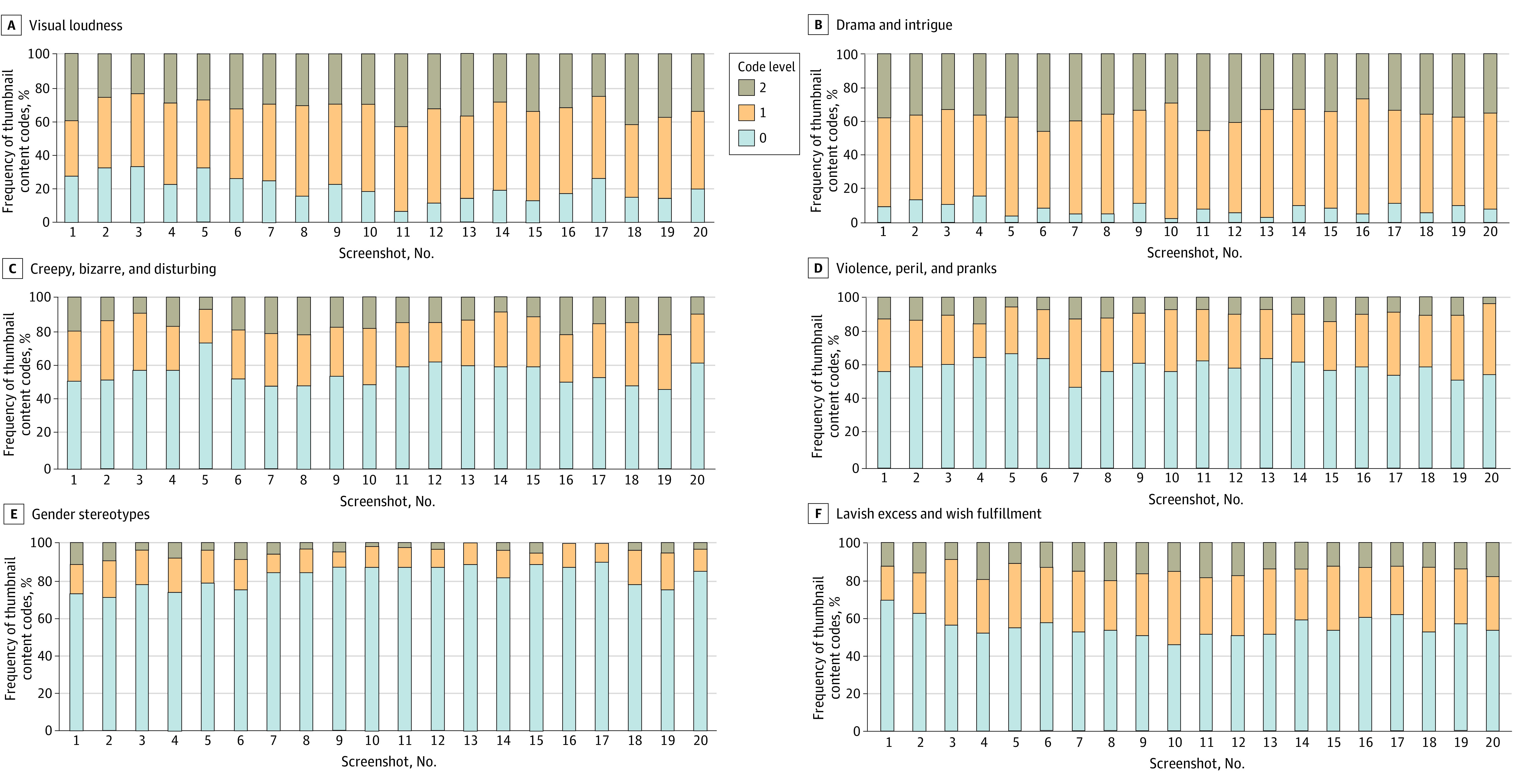
Frequency of Thumbnail Content Codes by Sequence of Screenshot (1-20), Averaged Over All 12 Search Terms The figure shows the frequency of content codes by the sequence of screenshot (1-20) for visual loudness (A), drama and intrigue (B), creepy, bizarre, and disturbing (C), violence, peril, and pranks (D), gender stereotypes (E), and lavish excess and wish fulfillment (F). Regarding coding level, level 0 indicated absent; level 1, present to a lesser degree or latent and implied; and level 2, clearly present.

## Discussion

In this cross-sectional study of VSP thumbnail image content recommended after popular searches, we described attention-capture designs that are highly prevalent in the visual stimuli children might see on recommendation feeds. Many of these features included age-inappropriate content such as gender stereotypes, horror characters, or violence. Contrary to our hypothesis, the prevalence of most codes did not increase over time when following recommendations over 20 sequential engagements; prevalence only increased slightly for visual loudness and decreased for gender stereotypes. It is possible that prevalence of attention-capture design was already high enough at the outset that random engagement with recommended videos did not change their prevalence in a substantial manner.

These findings represent novel evidence about what a child may encounter when using platforms that offer thumbnail-based arrays of recommended content. We focused on VSPs because they comprise the highest proportion of younger children’s media exposure,^1^ but our findings have relevance for social media platforms that use thumbnails and automated recommendations. The large amount of user-generated content on VSPs and social media means that they can be a source of positive or negative media experiences for children,^4,6,19^ much of which is shaped by automated recommendations. Our findings are consistent with prior work that identified age-inappropriate content on video platforms, both through automated detection^7^ and via parent report.^4^

Our results also suggest that content creators use clickbait-type features to encourage views, which can translate to increased monetization through advertising impressions or selling of merchandise.^6^ Our coding scheme contributes to the growing research on how digital designs aim to capture and sustain user attention,^18^ demonstrating how certain heuristics, such as children’s fascination with scary or violent content, sexual images, or luxury are leveraged for engaging click-through. Content creators create thumbnails using VSP tools or graphic design software and some VSPs allow content creators to A/B test their thumbnails (ie, release 2 different versions and keep the thumbnail that generates the most engagement). When creators realize that thumbnails with certain characteristics tend to be more successful in generating interest in their content, they may use these tactics repeatedly. Scholars have noted how platform algorithms can shape the aesthetic agendas of content creators, who then release content they think is more likely to trend.^20^

Only one other study has examined VSP algorithms and children’s content. Papadamou et al^7^ followed toddler-relevant searches (eg, *Peppa Pig*) for 10 hops and found a 3.5% likelihood that inappropriate videos appear in recommendation feeds. This relatively low incidence suggests that platforms may have successfully labeled and filtered content that is clearly directed to the youngest viewers. However, video game, influencer, and humorous content is more likely to be considered in a general audience category and, thus, have fewer constraints on automated recommendations. In adolescents and adults, algorithmic recommendations on social media platforms have been implicated in amplifying racially and ethnically insensitive and problematic content.^15,21^ Our results likely differ because we selected the next video at random, not based on a particular thumbnail feature such as horror or sexualized images, which might have led to more concentrated recommendations of problematic content over time.

### Limitations

This study has relevance for clinical and policy debates regarding children’s use of social media and VSPs that employ attention-capture designs and algorithmic recommendations, but also has several limitations. First, we only analyzed thumbnail content and did not watch full videos. However, this approach is important because in the current digital environment, recommendation grids and thumbnails represent decision points in which attention-capture designs shape children’s subsequent media experiences. A child may conceptualize recommended videos as implicitly endorsed by the platform or aligned with the child’s identity (ie, feeds that say, “for you”), which may influence the child’s norms about video content. This is consistent with priming theory,^22^ which suggests that digital stimuli, such as thumbnails with extreme features, might influence the way a child makes sense of the video they subsequently watch. Over time, with repeated exposure to such content, cultivation theory posits that the child may start to internalize norms or attitudes that are presented in media,^23^ which in this case might include normalization of gender stereotypes, materialism, or violence. In addition, young children’s exposure to negative content is associated with outcomes such as poor sleep^9^ and externalizing behavior,^10^ so methods for preventing such exposure are needed.

Another limitation of this study is that it captured VSP recommendations at one particular point in time (ie, search terms from 2020 or data collected in January 2022). Cultural trends, like the violent series *Squid Game*, were apparent in our screenshots, which can be expected to differ over time. Therefore, replication of our coding scheme within other data sets collected on different platforms is needed in addition to larger automated coding and data collection approaches.^15^ The platform we researched may have changed its algorithm since data were collected and our random choice of thumbnail may have limited the degree to which we would go down the rabbit hole. Future research is needed that examines how children react to attention-capture designs, how following recommendation feeds shifts children’s viewing preferences, and how recommendations differ from child to child based on their individual characteristics.

## Conclusions

Children spend a substantial portion of their media experiences on large platforms that distribute user-generated content of variable quality via automated recommendations. It is important to understand how the design of these platforms—both the user interface and the underlying algorithms—shapes children’s opportunities and risks. Large platforms with billions of hours of content cannot perform human review on every video before making viewing recommendations; therefore, parents and children will need to be aware of the likelihood of discovering inappropriate content and develop strategies for avoiding it. Alternatively, parents might choose video platforms where content is curated and reviewed by experts. More research on children’s experiences with algorithms and digital platforms is needed to inform clinical practice and policy.

## Notes

Supplement 1. **eTable 1.** Frequency (%) of Thumbnail Feature Codes by Search Term **eTable 2.** Description and Examples of Thumbnail Content in Recommended Videos

Supplement 2.
Data Sharing Statement

## References

[1] Rideout V, Robb MB. The common sense census: media use by kids age zero to eight. Common Sense Media. November 17, 2020. Accessed April 17, 2020. https://www.commonsensemedia.org/research/the-common-sense-census-media-use-by-kids-age-zero-to-eight-2020

[2] Radesky JS, Seyfried JL, Weeks HM, Kaciroti N, Miller AL. Video-sharing platform viewing among preschool-aged children: differences by child characteristics and contextual factors. Cyberpsychol Behav Soc Netw. 2022;25(4):230-236. 35426731 10.1089/cyber.2021.0235 PMC9051865

[3] Dixon SJ. Media usage in an internet minute as of April 2023. Statista. January 2, 2024. Accessed April 17, 2024. https://www.statista.com/statistics/195140/new-user-generated-content-uploaded-by-users-per-minute/

[4] Auxier BA, Anderson M, Perrin A, Turner E. Parenting children in the age of screens. Pew Research Center. July 28, 2020. Accessed January 19, 2023. https://www.pewresearch.org/internet/2020/07/28/parenting-children-in-the-age-of-screens/

[5] Tahir R, Ahmed F, Saeed H, Ali S, Zaffar F, Wilson C. Bringing the kid back into YouTube kids: Detecting inappropriate content on video streaming platforms. IEEE; 2019:464-469. doi:10.1145/3341161.3342913

[6] Radesky JS, Schaller A, Yeo S, Weeks HM, Robb MB. Young kids and YouTube: how ads, toys, and games dominate viewing. Common Sense Media. November 17, 2020. Accessed April 17, 2024. https://www.commonsensemedia.org/research/young-kids-and-youtube-how-ads-toys-and-games-dominate-viewing

[7] Papadamou K, Papasavva A, Zannettou S, Disturbed YouTube for kids: characterizing and detecting inappropriate videos targeting young children-proceedings from the fourteenth international AAAI conference on web and social media. Association for the Advancement of Artificial Intelligence. May 25, 2020. Accessed April 17, 2024. https://ojs.aaai.org/index.php/ICWSM/article/view/7320/7174

[8] Rollins D, Bridgewater E, Munzer T, . Who is the “you” in YouTube? Missed opportunities in race and representation in children’s YouTube videos. Common Sense Media. June 14, 2022. Accessed April 17, 2024. https://www.commonsensemedia.org/research/who-is-the-you-in-youtube-missed-opportunities-in-race-and-representation-in-childrens-youtube-videos

[9] Garrison MM, Liekweg K, Christakis DA. Media use and child sleep: the impact of content, timing, and environment. Pediatrics. 2011;128(1):29-35. doi:10.1542/peds.2010-3304 21708803 PMC3124101

[10] Tomopoulos S, Dreyer BP, Valdez P, . Media content and externalizing behaviors in Latino toddlers. Ambul Pediatr. 2007;7(3):232-238. doi:10.1016/j.ambp.2007.02.004 17512884

[11] Coyne SM, Holmgren HG, Shawcroft JE, . ABCs or attack-boom-crash? a longitudinal analysis of associations between media content and the development of problematic media use in early childhood. Technol Mind Behav. 2022;3(4). doi:10.1037/tmb0000093 37908683 PMC10617637

[12] Perelli A. How much money YouTubers make, according to dozens of creators. Business Insider. Updated January 3, 2024. Accessed April 17, 2024. https://www.businessinsider.com/how-much-do-youtubers-make

[13] Zhang S, Aktas T, Luo J. Mi YouTube es su YouTube? Analyzing the cultures using YouTube thumbnails of popular videos. Paper presented at: the 2021 IEEE International Conference on Big Data; December 15, 2021; Orlando, FL. Accessed April 17, 2024. https://ieeexplore.ieee.org/document/9672037

[14] Zannettou S, Chatzis S, Papadamou K, Sirivianos M. The good, the bad and the bait: Detecting and characterizing clickbait on YouTube. Paper presented at: 2018 IEEE Security and Privacy Workshops Conference; May 24, 2018; San Francisco, CA. doi:10.1109/SPW.2018.00018

[15] Yesilada M, Lewandowsky S. Systematic review: YouTube recommendations and problematic content. Internet Policy Rev. 2022;11(1):1652. doi:10.14763/2022.1.1652 36466439 PMC7613872

[16] Obermeyer Z, Powers B, Vogeli C, Mullainathan S. Dissecting racial bias in an algorithm used to manage the health of populations. Science. 2019;366(6464):447-453. doi:10.1126/science.aax2342 31649194

[17] Vlasceanu M, Amodio DM. Propagation of societal gender inequality by internet search algorithms. Proc Natl Acad Sci U S A. 2022;119(29):e2204529119. doi:10.1073/pnas.2204529119 35858360 PMC9304000

[18] Monge Roffarello A, Lukoff K, De Russis L. Defining and identifying attention capture deceptive designs in digital interfaces. Association for Computing Machinery. April 2023. Accessed April 17, 2024. https://dl.acm.org/doi/full/10.1145/3544548.3580729

[19] Rideout V, Peebles A, Mann S, Robb M. The common sense census: media use by tweens and teens. Common Sense Media. March 9, 2022. Accessed April 17, 2024. https://www.commonsensemedia.org/research/the-common-sense-census-media-use-by-tweens-and-teens-2021

[20] Balanzategui J. ‘Disturbing’ children’s YouTube genres and the algorithmic uncanny. New Media Soc. 2023;25(12):3521-3542. doi:10.1177/14614448211049264

[21] Williams D, Farthing R, McIntosh A. Surveilling young people online: an investigation into TikTok’s data processing practices. Reset Australia. July 2021. Accessed April 17, 2024. https://au.reset.tech/uploads/resettechaustralia_policymemo_tiktok_final_online.pdf

[22] Roskos-Ewoldsen DR, Klinger MR, Roskos-Ewoldsen B. Media priming: a meta-analysis. In: Press RW, Gayle BM, Burrell N, Allen M, Bryant J, eds. Mass Media Effects Research: Advances Through Meta-Analysis. Lawrence Erlbaum Associates Publishers; 2007:53-80.

[23] Gerbner G, Gross L, Morgan M, Signorielli N, Shanahan J. Growing up with television Cultivation processes. In: Bryant J, Zillman D, eds. Media Effects: Advances in Theory and Research. 2nd ed. Lawrence Erlbaum Associates Publishers; 2002:43-67.

[24] Hardwick J. Top YouTube searches. Ahrefsblog. Accessed Updated January 1, 2021. April 17, 2024. https://ahrefs.com/blog/top-youtube-searches/

