# Supplementary material for: Algorithmic Content Recommendations on a Video-Sharing Platform Used by Children
Source: JAMA Netw Open. 2024 May 29;7(5):e2413855. doi: 10.1001/jamanetworkopen.2024.13855 (PMC11137630; doi:10.1001/jamanetworkopen.2024.13855)
Supplement: Supplement 1. — eTable 1. Frequency (%) of Thumbnail Feature Codes by Search Term eTable 2. Description and Examples of Thumbnail Content in Recommended Videos [file jamanetwopen-e2413855-s001.pdf]

Supplemental Online Content

Radesky J, Bridgewater E, Black C, et al. Algorithmic content recommendations on a video-sharing platform used by children. *JAMA Netw Open*. 2024;7(5):e2413855. doi:10.1001/jamanetworkopen.2024.13855

eTable 1. Frequency (%) of Thumbnail Feature Codes by Search Term

eTable 2. Description and Examples of Thumbnail Content in Recommended Videos

This supplemental material has been provided by the authors to give readers additional information about their work.

© 2024 Radesky J et al. *JAMA Network Open.*

eTable 1. Frequency (%) of Thumbnail Feature Codes by Search Term

Feature Codes	DanTDM	FGTeev	Flamingo	Fortnite	Memes	Minecraft	MrBeast	Pewdiepie	Roblox	Sml	Try not to laugh	Unspeakable
**Visual Loudness**												
**0**	42 (17.5)	26 (10.8)	44 (18.3)	61 (25.4)	68 (28.3)	34 (14.2)	34 (14.2)	91 (37.9)	64 (26.7)	10 (4.2)	72 (30.0)	56 (23.3)
**1**	104 (43.3)	119 (49.6)	113 (47.1)	130 (54.2)	105 (43.8)	128 (61.3)	147 (61.3)	99 (41.3)	128 (53.3)	49 (20.4)	89 (37.1)	141 (58.8)
**2**	94 (39.2)	95 (39.6)	83 (34.6)	49 (20.4)	67 (27.9)	78 (24.6)	59 (24.6)	50 (20.8)	48 (20.0)	181 (75.4)	79 (32.9)	43 (17.9)
**Drama and Intrigue**												
**0**	17 (7.1)	12 (5.0)	21 (8.8)	22 (9.2)	30 (12.5)	12 (5.0)	16 (6.7)	25 (10.4)	8 (3.3)	22 (9.2)	33 (13.8)	26 (10.8)
**1**	121 (50.4)	153 (63.8)	100 (41.7)	139 (57.9)	133 (55.4)	135 (56.3)	142 (59.2)	145 (60.4)	115 (47.9)	152 (63.3)	129 (53.8)	142 (59.2)
**2**	102 (42.5)	75 (31.3)	119 (49.6)	79 (32.9)	77 (32.1)	93 (38.8)	82 (34.2)	70 (29.2)	117 (48.8)	66 (27.5)	78 (32.5)	72 (30.0)
**Creepy, Bizarre, and Disturbing**												
**0**	135 (56.3)	125 (52.1)	50 (20.8)	194 (80.8)	85 (35.4)	118 (49.2)	161 (67.1)	169 (70.4)	178 (74.2)	119 (49.6)	111 (46.3)	152 (63.3)
**1**	59 (24.6)	81 (33.8)	107 (44.6)	33 (13.8)	79 (32.9)	82 (34.2)	53 (22.1)	42 (17.5)	46 (19.2)	113 (47.1)	87 (36.3)	60 (25.0)
**2**	46 (19.2)	34 (14.2)	83 (34.6)	13 (5.4)	76 (31.7)	40 (16.7)	26 (10.8)	29 (12.1)	16 (6.7)	8 (3.3)	42 (17.5)	28 (11.7)
**Violence, Peril, and Pranks**												
**0**	125 (52.1)	134 (55.8)	95 (39.6)	158 (65.8)	140 (58.3)	135 (56.3)	137 (57.1)	153 (63.8)	148 (61.7)	189 (78.8)	151 (62.9)	145 (60.4)
**1**	91 (37.9_	98 (40.8)	66 (27.5)	74 (30.8)	76 (31.7)	76 (31.7)	94 (39.2)	68 (28.3)	67 (27.9)	44 (18.3)	62 (25.8)	75 (31.3)
**2**	24 (10.0)	8 (3.3)	79 (32.9)	8 (3.3)	24 (10.0)	29 (12.1)	9 (3.8)	19 (7.9)	25 (10.4)	7 (2.9)	27 (11.3)	20 (8.3)
**Gender Stereotypes**												
**0**	202 (84.2)	195 (81.3)	176 (73.3)	186 (77.5)	205 (85.4)	216 (90.0)	221 (92.1)	206 (85.8)	139 (57.9)	187 (77.9)	196 (81.7)	226 (94.2)
**1**	27 (11.3)	33 (13.8)	55 (22.9)	39 (16.3)	23 (9.6)	17 (7.1)	15 (6.3)	28 (11.7)	63 (26.3)	40 (16.7)	33 (13.8)	14 (5.8)
**2**	11 (4.6)	12 (5.0)	9 (3.8)	15 (6.3)	12 (5.0)	7 (2.9)	4 (1.7)	6 (2.5)	38 (15.8)	13 (5.4)	11 (4.6)	0 (0)
**Lavish Excess and Wish Fulfillent**												
**0**	137 (57.1)	125 (52.1)	185 (77.1)	106 (44.2)	169 (70.4)	142 (59.2)	94 (39.2)	155 (64.6)	102 (42.5)	101 (42.1)	163 (67.9)	115 (47.9)
**1**	65 (27.1)	88 (36.7)	47 (19.6)	83 (34.6)	53 (22.1)	63 (26.3)	107 (44.6)	60 (25.0)	79 (32.9)	81 (33.8)	52 (21.7)	82 (34.2)
**2**	38 (15.8)	27 (11.3)	8 (3.3)	51 (21.3)	18 (7.5)	35 (14.6)	39 (16.3)	25 (10.4)	59 (24.6)	58 (24.2)	25 (10.4)	43 (17.9)

© 2024 Radesky J et al. *JAMA Network Open.*

eTable 2. Description and Examples of Thumbnail Content in Recommended Videos*

Feature Name	Description and Examples
**Visual Loudness**	Attention-grabbing visual design that used perceptually salient features such as:<br>• bright/highly saturated colors: https://www.youtube.com/watch?v=zxYjTTXc-J8; https://www.youtube.com/watch?v=hJTYP7MnuHE<br>• high density of characters: https://www.youtube.com/watch?v=4VfO5Jj4a-c<br>• dark colors or high contrast: https://www.youtube.com/watch?v=8J_zM1zYsHg&t=55s&pp=ygUGZGFudGRt<br>• text in large letters with lots of exclamation points: https://www.youtube.com/watch?v=9vxXAdp78S8&pp=ygUDc21s or large faces with extreme facial expressions: https://www.youtube.com/watch?v=nM89Wl03Q4g&pp=ygUSbWluZWNyYWZ0IDEwMCBkYXlz
**Drama and Intrigue**	Images that conveyed a shocking, dramatic, or outrageous message that invites click-through to find out what is going on. Includes:<br>• manufactured conflict (e.g., "VS" thumbnails): https://www.youtube.com/watch?v=zQII_q_ycpE<br>• facial expressions that are extreme, shocked or disgusted: https://www.youtube.com/watch?v=DphMZBbmjvw&pp=ygUSbWluZWNyYWZ0IDEwMCBkYXlz<br>• dramatic situations: https://www.youtube.com/watch?v=hxwpkM5w3Cc<br>• images that are intentionally vague, confusing, or altered or strange in a way that aims to arouse curiosity: https://www.youtube.com/watch?v=rKLk0JUS4TE&pp=ygUQdHJ5IG5vdCB0byBsYXVnaA%3D%3D
**Lavish Excess and Wish Fulfillment**	Depiction of luxury items such as:<br>• cars, jewelry, houses: https://www.youtube.com/watch?v=KSKJKLmAqpI<br>• large amounts of food: https://www.youtube.com/watch?v=UkZH3a79tOM or https://www.youtube.com/watch?v=_dfjMq3ZL4w&pp=ygUUdW5zcGVha2FibGUgZm9ydG5pdGU%3D<br>• lots of money (any depiction of a large amount of currency, even if a negative value): https://www.youtube.com/watch?v=Vl3swga-Xrk&pp=ygUfbXJiZWFzdCBnaWFudCBkaWFtb25kIG9yIDEwMDAwIA%3D%3D<br>• Includes objects that viewers might not be able to experience in real life - such as watching an extensive Minecraft world being built, lots of toys/objects on display, unboxing, or

© 2024 Radesky J et al. *JAMA Network Open.*

	expensive technology or items: https://www.youtube.com/watch?v=kxmRiC6O73A&pp=ygUacHJlc3RvbiBtaW5lY3JhZnQgZGlhbW9uZHM%3D or https://www.youtube.com/watch?v=hK6A_ebUKrM&pp=ygUWdHlwaWNhbCBnYW1lciBmb3J0bml0ZQ%3D%3D
**Creepy, Bizarre, and Disturbing**	Thumbnails include elements such as:<br>• Odd, distorted images (e.g., cartoon faces, odd juxtapositions like faces on suitcases): https://www.youtube.com/watch?v=9S4zsQNgxEI<br>• depictions of frightening characters or objects (e.g., *Venom*, *Squid Game*: https://www.youtube.com/watch?v=0e3GPea1Tyg; https://www.youtube.com/watch?v=8J_zM1zYsHg<br>• skeletons, monsters, coffins: https://www.youtube.com/watch?v=kTyOLGzEb7M<br>• Images also seemed to elicit disgust or leverage attraction to horror content: https://www.youtube.com/watch?v=HIoGNh5evYg
**Violence, Peril, and Pranks**	Thumbnails depict:<br>• Gore, injury, violence: https://www.youtube.com/watch?v=x03CdpPw6Lc or https://www.youtube.com/watch?v=egEicW9Q1mA&pp=ygUdc3Vydml2YWwgdGhlIHNwb25nZWJvYiBraWxsZXI%3D<br>• people appearing to prank one another or do dangerous activities: https://www.youtube.com/watch?v=OYWA0W3WWy4&pp=ygUGcHJhbmtz<br>• appearance of weapons or dead bodies: https://www.youtube.com/watch?v=8SgEKVCErQE<br>• dangerous substances like lava: https://www.youtube.com/watch?v=VtbqyPdxhvo<br>• 'challenges' that are perilous, references to death, or a threat of injury (e.g., car crashes): https://www.youtube.com/watch?v=_qVIBVNT41I&pp=ygUSZmxhbWluZ28gdHJhcCBub29i
**Gender Stereotypes**	Sexual objectification to attract click-through, including:<br>• depictions of male or female exaggerated bodies: https://www.youtube.com/watch?v=X1R7Zu8-ero<br>• sexual content or innuendos: https://www.youtube.com/watch?v=UD7bo--VmGA&pp=ygUQYnJvb2toYXZlbiBvZGVycw%3D%3D<br>• idealized depictions of bodies in video games (e.g., Lara Croft bodies in Fortnite or comics; men with "6-pack" abs): https://www.youtube.com/watch?v=IjfOOK1M4go&pp=ygUPc3BpZGVybWFuIEdUQSA1

*Search terms included: PewDiePie, Fortnite, DanTDM, Minecraft, MrBeast, FGTeeV, Flamingo, Memes, Unspeakable, Try not to laugh, Roblox, and SML

© 2024 Radesky J et al. *JAMA Network Open.*
