# Supplementary material for: Algorithmic Content Recommendations on a Video-Sharing Platform Used by Children
Source: JAMA Netw Open. 2024 May 29;7(5):e2413855. doi: 10.1001/jamanetworkopen.2024.13855 (PMC11137630; doi:10.1001/jamanetworkopen.2024.13855)
Supplement: Supplement 2. — Data Sharing Statement [file jamanetwopen-e2413855-s002.pdf]

Data Sharing Statement

Radesky. Algorithmic Content Recommendations on a Video-Sharing Platform Used by Children. *JAMA Netw Open*. Published May 29, 2024.
doi:10.1001/jamanetworkopen.2024.13855

Data
Data available: No

Additional Information
Explanation for why data not available: I do not have a public repository to make it available, but am happy to provide the data (not human subjects) to others on request.
